# Unveiling the Genetic Complexity: A Case of 46 XY Disorder of Sex Development Linked to Chromosome 9 Inversion and Its Multidisciplinary Management

**DOI:** 10.7759/cureus.79189

**Published:** 2025-02-17

**Authors:** Punith Jain R, Suryaram Aravind, Velmurugan Palaniyandi, Hariharasudhan Sekar, Sriram Krishnamoorthy

**Affiliations:** 1 Department of Urology and Renal Transplantation, Sri Ramachandra Institute of Higher Education and Research, Chennai, IND

**Keywords:** ambiguous genitalia, chromosome 9 inversion, infertility, karyotype, sex differentiation, sry gene

## Abstract

This is a case report of a five-year-old patient with a rare 46 XY DSD (disorder of sex differentiation) due to chromosome 9 inversion. DSD involves a wide spectrum of genetic, gonadal, and anatomical abnormalities that are linked to complex clinical conditions of 46 XY DSD in the presence of a normal *SRY* gene. Ambiguous genitalia due to chromosome 9 inversion is a rare occurrence triggering further research options. The patient presented with ambiguous genitalia, difficulty in urination, and inguinal swelling. Despite a typical *SRY* gene, the inversion likely disrupted other crucial genes impacting sexual and neurological development. Laparoscopic-assisted total urogenital mobilization was performed on the patient, which resulted in improved urinary function and cosmetic outcomes. This case illustrates that genetic influences in DSD are complex, implying the need for a multidisciplinary approach to diagnosis and management. It outlines that chromosome 9 inversion should always be taken into consideration when a normal *SRY* gene is present, as observed in our case.

## Introduction

Disorder of sex differentiation (DSD) can result from mutations in sex-determining genes to androgen receptor defects [1]. This wide spectrum of genetic, gonadal, and anatomical abnormalities is linked to complex clinical conditions of 46 XY DSD in the presence of a normal *SRY* gene, indicating there can be potential alternative genetic or epigenetic mechanisms involving the alterations in chromosome 9 [2,3]. The pericentric chromosome 9 inversion has been reported in the literature supporting reproductive, cognitive, and developmental abnormalities [4,5]. However, the precise role of chromosome 9 inversion in sex reversal remains unclear to date. Hence, this case report highlights its implications in sex determination and opens a new area for research, considering this phenomenon as a potential reason for sex reversal [6].

## Case presentation

History

A five-year-old child presented to our hospital for evaluation following the observation of ambiguous genitalia, difficulty in passing urine, and swelling in both groins along with pain. The child has been having urinary difficulties for the past few months and has been experiencing symptoms of swelling and pain in the groin region for the past two weeks. Apart from this, the child exhibited notable developmental issues such as microcephaly, global developmental delay, and a history of repeated seizures.

Examination and imaging

**Figure 1:**
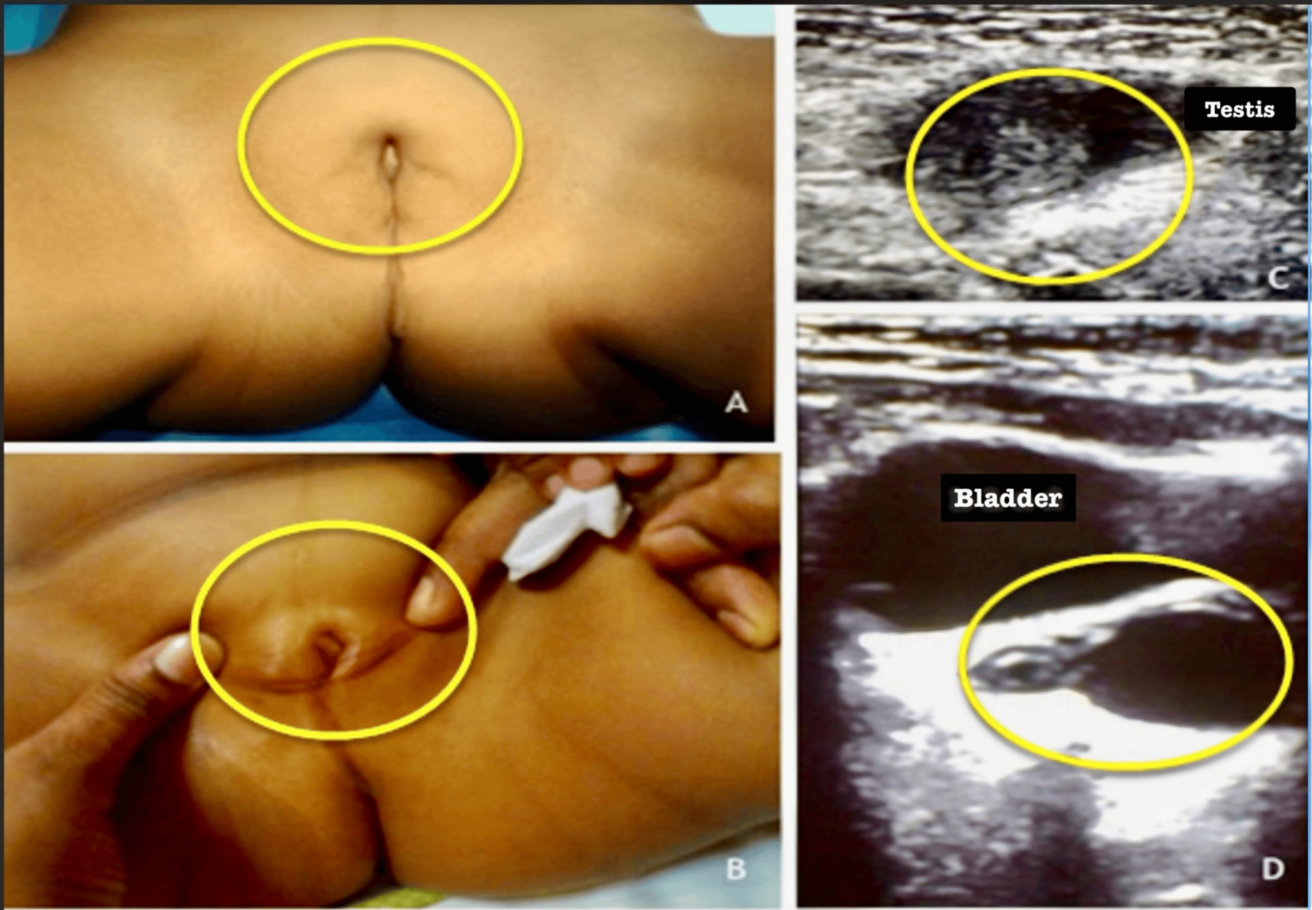
Gross examination of the genitalia (A,B) and ultrasound imaging of the abdomen and pelvis (C,D) All the regions of importance have been marked for better understanding and interpretation. They exhibit the following: (A) genitalia with partially fused labia majora and absent vulva, (B) an underdeveloped clitoris with a single urogenital sinus opening, (C) gonads with testicular echoes at the deep ring, and (D) bladder and rudimentary uterus with vagina (fluid-filled tube).

Bilateral inguinal hernia and palpable gonads were identified in the inguinal regions. The external genital region showed a partially formed clitoris, a single urogenital sinus opening (Figure 1A), and the partially fused labia majora, together with an absence of labia minora and vulva (Figure 1B). An abdominal ultrasound revealed gonads at the deep inguinal ring, which corresponds to testicular echoes (Figure 1C). In addition, there was a fluid-filled, hypo-echoic tube-like structure behind the urinary bladder, pointing to a rudimentary uterus and possibly a vaginal remnant (Figure 1D). These findings suggested an aberrant formation of the genitals, prompting further investigations.

Karyotype

**Figure 2:**
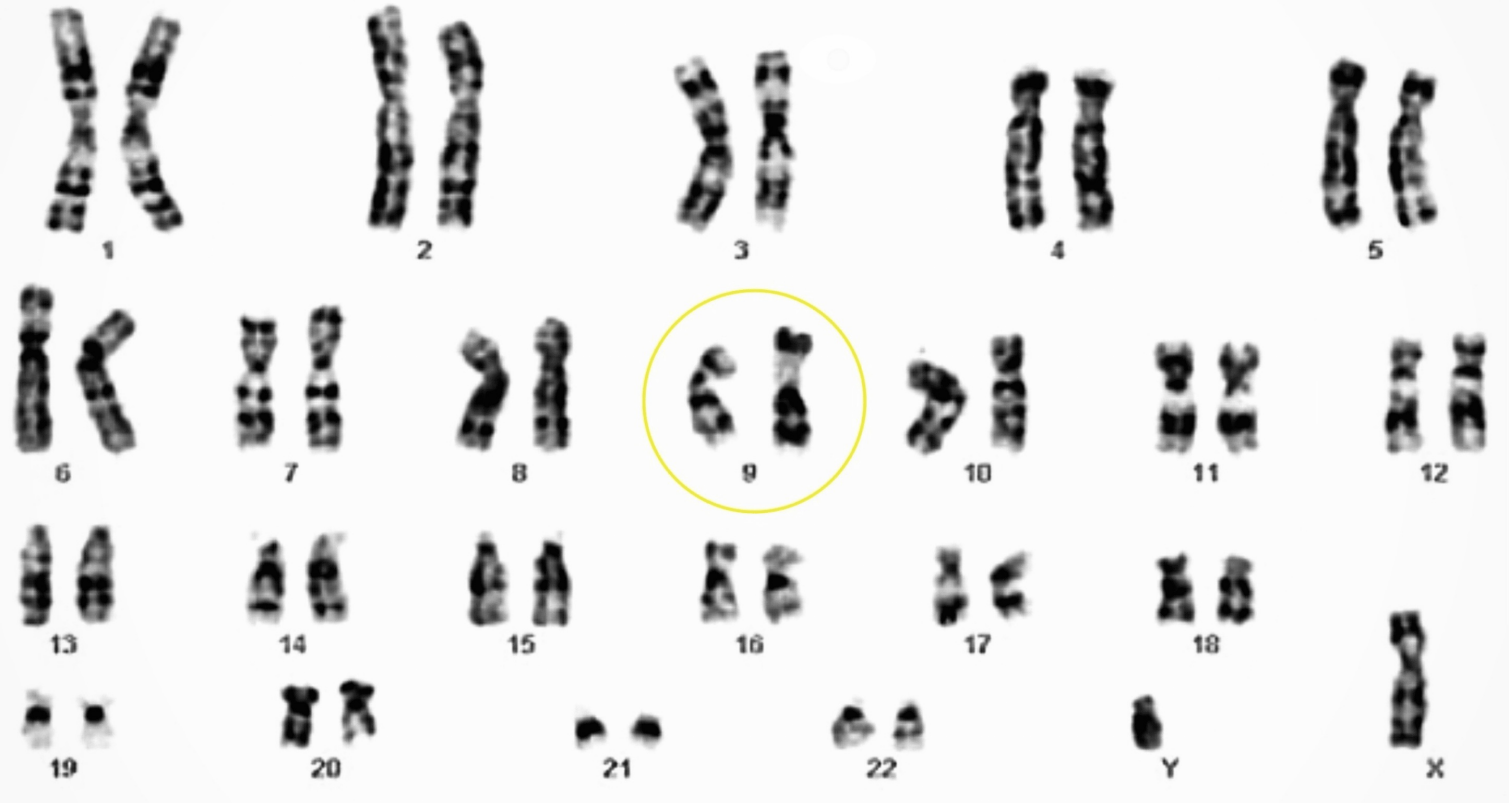
Karyotyping It shows 46 XY with inversion of chromosome 9 and no deletion of the *SRY* gene.

Karyotyping (Figure 2) revealed a 46 XY genetic makeup, ruling out an *SRY* gene deletion and indicating a chromosome 9 inversion. Blood testing revealed normal serum cortisol, DHEAS (dehydroepiandrosterone sulfate), serum calcium, phosphate, electrolytes, serum urea, creatinine, liver function, and thyroid function tests.

Other investigations

**Figure 3:**
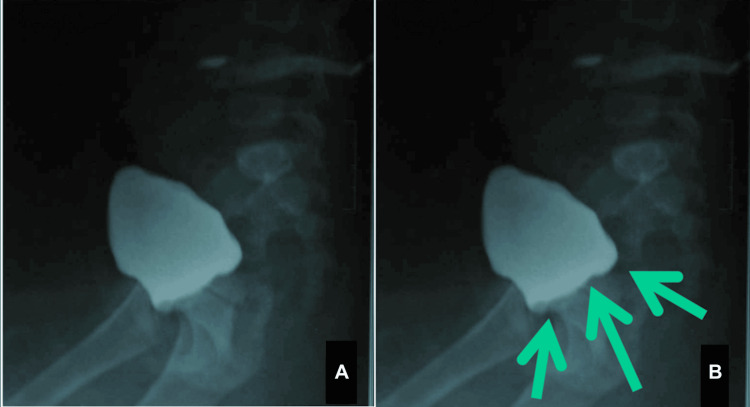
Suprapubic cystogram The region of importance is marked for better understanding and interpretation: (A) a cystogram with an elevated bladder base and (B) an indentation caused by the Müllerian structures (green arrows).

Catheterization was attempted and failed, and a genitogram could not be performed, but a suprapubic cystogram was done, showing an elevated bladder base (Figure 3A), likely due to the indentation of Müllerian structures (Figure 3B). Based on the clinical, karyogram, and radiological findings, the child was diagnosed with 46 XY DSD with sex reversal, probably secondary to chromosome 9 inversion.

Treatment

**Figure 4:**
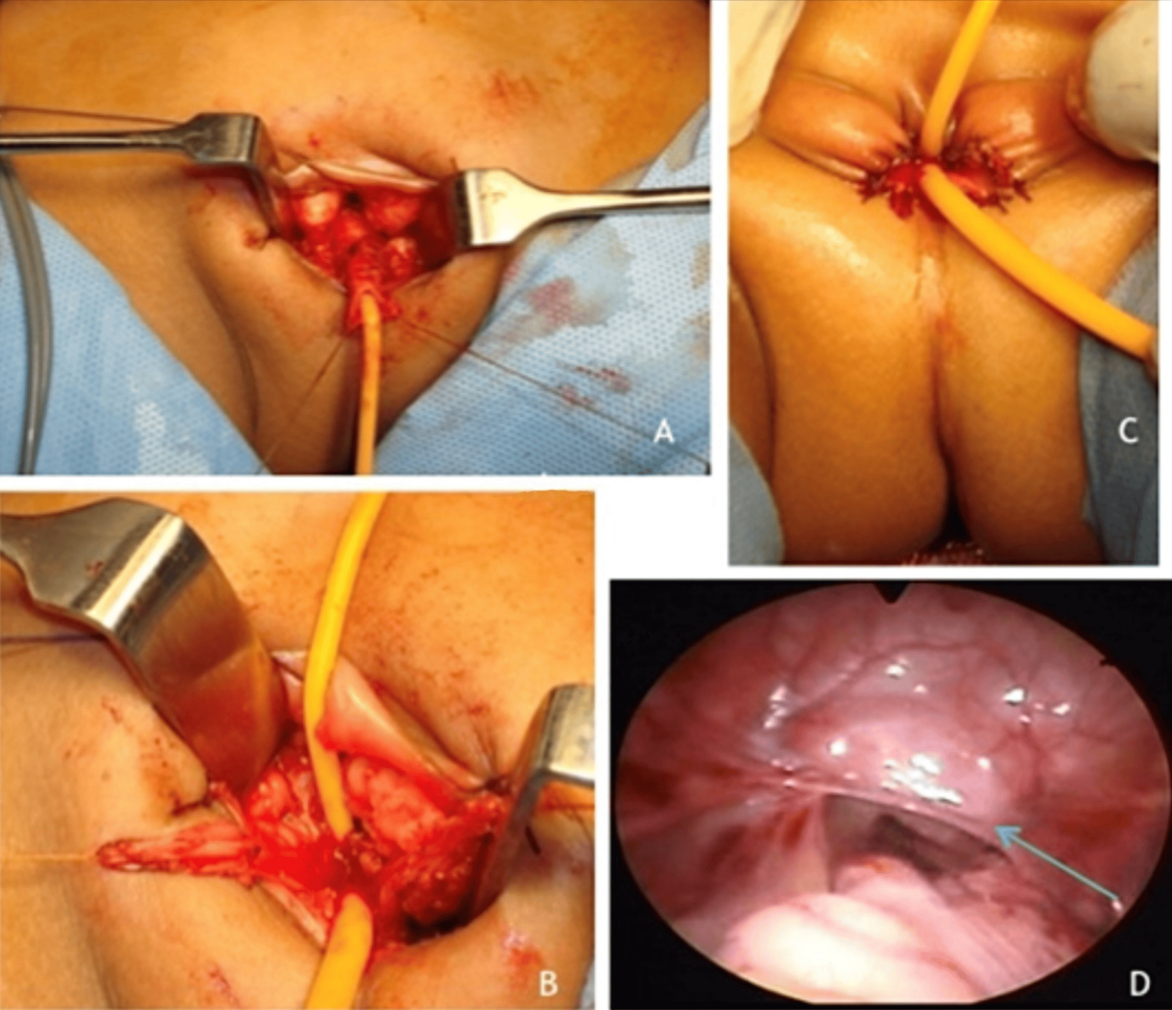
Steps of total urogenital mobilization (A) Mobilization of the urethra; (B) reconstruction of vaginal introitus and urethra (two separate openings); (C) end result (after hemostasis and closure); (D) laparoscopic view of the testis (green arrow).

The child underwent laparoscopic-assisted total urogenital mobilization (Figures 4A-C show steps of urogenital mobilization; Figure 4D shows the testis identified and mobilized laparoscopically). Intraoperatively, bilateral testes were identified at the level of the deep inguinal ring, and persistent Müllerian structures, including a rudimentary uterus and vagina, were observed. These structures are typically absent in males, as the Müllerian ducts regress during normal male development.

Outcomes and follow-up

Following the procedure, the wound is healthy with the two separate passages (urogenital). Postoperatively, the child is experiencing satisfactory cosmetic outcomes and is passing urine without difficulty, and follow-up visits showed no signs of urinary tract infections or complications. The child is currently kept on follow-up, and parents were counseled regarding the possible complications (risk of gonadal tumors, subfertility or infertility, and neurocognitive and psychosocial disturbances).

## Discussion

Sex differentiation is a tightly regulated phenomenon, with the *SRY* gene playing a pivotal role in initiating the process of testicular differentiation, but an alternative pathway influencing gonadal development is observed in cases with normal *SRY* gene function [7]. Genetic and epigenetic concepts associated with chromosome 9 inversion leading to congenital anomalies, infertility, and recurrent pregnancy loss have been widely described in the literature [8,9].

Genes involved in gonadal development contributing to reproductive dysfunction associated with chromosome 9 inversion have been reported in several studies [10]. The key sex-determining genes, such as *SOX9*, *DAX1*, and *WT1*, have their regulatory elements disrupted, altering their expression and leading to sex reversal. This phenomenon has been hypothesized in the literature, with rarity occurring when the SRY gene is present [11]. Further research into chromosomal rearrangement detecting few contributory evidence toward DSD can explore the potential involvement of chromosome 9 inversion in cases of XY females [12].

Historically, chromosome 9 inversions were considered benign, but extensive associations have been identified with cognitive, reproductive, and developmental processes. These associations have prompted consideration of the potential pathologies related to chromosome 9 inversion [13]. The coexistence of trisomy 22 with chromosome 9 inversion can be misleading, where in one such report, the real phenotypic alterations were attributed to mosaic trisomy 22. Can it be an inversion of chromosome 9, especially when there is a mosaic trisomy 22 with masquerading phenotypic variation [14]?

Large clinical profile studies [15] on 46 XY disorders have detailed several genetic alterations as potential triggers during differentiation. Understanding the molecular and genetic aspects of chromosome 9 pathologies in the future with next-generation sequencing and targeted sequencing shall focus on sex reversal in 46 XY DSD potentially due to chromosome 9 inversion. Advanced genomic sequencing models such as whole-genome sequencing and transcriptomic analysis can elucidate the functional impact of the chromosome 9 alterations on the sex differentiation pathway.

Take-home points

Education and Awareness

This case falls within the scant but expanding body of literature associating chromosome 9 inversions with sex development disorders, a reminder of the potential association and the need for further research.

Surgical and Ethical Considerations

Decisions about surgical interventions in DSD patients should be based on their age, psychosocial needs, potential future health risks, and an ethical approach to counseling patients or families for long-term follow-up.

Complexity of Genetic Interactions

This case illustrates that even a rare inversion of chromosome 9 can cause anomalies in sex differentiation and neurological development.

Multidisciplinary Management

This case is an example of how coordination of care (endocrinology, genetics, urology, and psychosocial support) leads to better outcomes for patients.

## Conclusions

Genetic influences in DSD are complex; the presence of a normal SRY gene in this case has made us wonder if chromosome 9 inversion is the reason for sex reversal. The outlines on investigation and management draw the need for a multidisciplinary approach to such conditions and understanding their importance. The role of surgical management should be reserved for selective candidates to achieve acceptable functional and cosmetic outcomes.
